# Assessing the Impact of an Instructional Module on Nurses’ Knowledge, Attitudes, and Practices for Preventing Hospital-Acquired Infections in Gautam Budh Nagar, Uttar Pradesh, India

**DOI:** 10.7759/cureus.94549

**Published:** 2025-10-14

**Authors:** Parul Gupta, Sanjana Gupta, Nazneen Arif, Prasidh Narayan Pathak, Rachna Khanna

**Affiliations:** 1 Department of Medical-Surgical Nursing (Critical Care), Kailash Institute of Nursing and Paramedical Science, Noida, IND; 2 Department of Oral and Maxillofacial Pathology and Microbiology, Institute of Technology and Science (ITS) Center for Dental Studies and Research, Ghaziabad, IND; 3 Division of Molecular Biology, National Institute of Cancer Prevention and Research, Indian Council of Medical Research, Noida, IND; 4 Department of Critical Care Medicine, Government Institute of Medical Science, Noida, IND; 5 Department of Obstetrics and Gynecology, Kailash Institute of Nursing and Paramedical Science, Noida, IND

**Keywords:** attitude, hospital acquired infection, instructional module, knowledge, practice, quality improvement and patient safety

## Abstract

Background

Healthcare-associated infections (HAIs) are a major cause of morbidity, mortality, and healthcare costs. Nurses in critical care units play a central role in infection prevention, but gaps in knowledge, attitudes, and practices (KAP) often compromise patient safety. Educational interventions provide a feasible strategy to strengthen infection control competencies.

Aim

This study evaluated the effectiveness of a structured instructional module in improving nurses’ KAP regarding HAI prevention in tertiary care hospitals of Gautam Budh Nagar district, Uttar Pradesh, India.

Materials and methods

A quasi-experimental pre-post design was conducted among 60 nurses recruited purposively from medical, surgical, neonatal/paediatric, and trauma ICUs across five tertiary care hospitals. Sample size was determined a priori using OpenEpi (Version 3.01), which indicated a minimum of 54 participants; 60 were enrolled to ensure robustness. Data were collected using a structured KAP questionnaire, validated by experts and pilot-tested for clarity and feasibility. Statistical analysis was performed using SPSS v26.0 (IBM Corp., Armonk, NY), applying paired t-test, chi-square test, and Fisher’s exact test, with significance set at p < 0.05.

Results

The instructional module significantly improved nurses’ knowledge, attitudes, and practices (p < 0.05), with moderate to large effect sizes observed. Gains were consistent across most domains, with some associations noted between outcomes and ICU type. The pilot testing confirmed tool feasibility and clarity.

Conclusion

A single, structured educational module effectively enhanced nurses’ HAI prevention competencies. Regular, context-specific training and integration into nursing curricula are essential to sustain improvements and reduce the HAI burden in high-demand healthcare settings.

## Introduction

Healthcare-associated infections (HAIs) pose a significant challenge in hospital settings, contributing to considerable morbidity and mortality [1-2]. HAIs arise when patients undergo treatment in healthcare environments, representing risks not only to the patients but also to healthcare workers exposed to various pathogens [3-4]. Nursing practices are pivotal in mitigating these infections, as nurses are essential to the healthcare delivery system and play a key role in quality improvement initiatives. The knowledge, attitudes, and practices of nurses have a direct impact on patient outcomes [5-6]. Research has shown that ongoing education enhances competencies related to infection prevention among nursing staff. Factors influencing nurses' knowledge levels include personal background, participation in training programs, and individual motivation [7-8]. Despite advancements in healthcare, HAIs remain a serious threat, leading to increased healthcare costs and preventable fatalities. Effective infection control necessitates robust education and a deep understanding of infection prevention practices, particularly in emergency departments that frequently face challenges like insufficient resources and high workloads [9-10]. This study proposes to evaluate an instructional module aimed at enhancing the knowledge, attitudes, and practices of nurses concerning the prevention of healthcare-associated infections (HAIs), specifically within the city of Gautam Budh Nagar, Uttar Pradesh, India.

## Materials and methods

Study design, setting and population

This quasi-experimental pre-test and post-test study evaluated the effectiveness of a structured instructional module in improving nurses’ knowledge, attitudes, and practices (KAP) regarding healthcare-associated infection (HAI) prevention. The study was conducted in five purposively selected tertiary care hospitals of Gautam Budh Nagar district, Uttar Pradesh, India, a rapidly urbanizing region within the National Capital Region (NCR). These hospitals were chosen for their established critical care services, high patient turnover, and relevance to infection control practices. Specialized units included medical, surgical, neonatal/pediatric, and trauma intensive care units (ICUs). The study population comprised registered nurses working in critical care units of the selected hospitals.

Eligibility criteria

Eligible participants were nurses who were (i) currently employed in ICUs and (ii) willing to participate in the study. Nurses working outside ICUs, those who declined participation, or those on leave during the data collection period were excluded.

Sample size and sampling procedure

The required sample size was calculated a priori using OpenEpi software (Version 3.01, www.OpenEpi.com) for paired pre-post designs. Parameters were derived from pilot study data and published evidence: a two-sided significance level of 0.05, 80% power (β = 0.20), an expected mean improvement of approximately 1.5 points in knowledge score, and a standard deviation of paired differences of about 3.0 (Cohen’s d ≈ 0.5-0.6). This indicated a minimum requirement of 54 participants. To strengthen robustness and account for attrition, 60 nurses were purposively recruited, with 30 each in the experimental and control groups.

Data collection tool and variable measurement

Data were collected using a structured, self-administered KAP questionnaire developed after a comprehensive literature review and validated by a panel of experts in infection control and nursing education.

The tool included (1) knowledge (15 multiple-choice questions (score range: 0-15; 1 point for correct responses); (2) attitude (10 items on a five-point Likert scale (score range: 10-50; higher scores indicate more positive attitudes)); and (3) practice (10 dichotomous items (score range: 0-10; Yes = 1, No = 0)).

Sociodemographic variables such as age, gender, qualification, years of experience, and ICU type were also collected. The tool was pilot-tested with six nurses (three control, three experimental), confirming clarity, feasibility, and completion within 15-20 minutes. Although internal consistency measures, such as Cronbach’s alpha, were not computed, this limitation was explicitly acknowledged.

Knowledge, attitude, and practice (KAP) assessment

The study was implemented in two phases. In the pilot phase (2023), six nurses were enrolled to pre-test the questionnaire, participate in a four-week instructional program (experimental group), and complete a post-test to refine the study design. In the main phase (2024), 60 nurses were recruited and divided into experimental and control groups (30 each). A pre-test was administered in July 2024. The experimental group then underwent a three-month instructional program (August-October 2024) based on a self-constructed educational module, while the control group received no intervention. Both groups completed the post-test in November 2024 using the same questionnaire. Data collection was facilitated through Google Forms to ensure accuracy, completeness, and efficiency.

Statistical analysis

Data analysis was conducted using SPSS® Version 26.0 (IBM Corp., Armonk, NY). Descriptive statistics (frequency, percentage, mean, and standard deviation) summarized demographic and KAP data. Associations between categorical variables were examined using chi-square and Fisher’s exact tests. Differences between pre- and post-test scores within groups were analyzed using paired t-tests. Statistical significance was set at p < 0.05.

## Results

Chi-square tests assessed associations between demographic variables and the levels of knowledge acquired outcomes in both pre-test and post-test phases. Results showed no significant associations between age, gender, or experience and outcomes (p > 0.05), with Cramér’s V effect sizes indicating limited significance (V = 0.00-0.286). However, a significant association was found between area of work and outcomes in the post-test phase (χ² (10) = 18.5, p = 0.035), with a moderate effect size (Cramér’s V = 0.390). Overall, while most demographic factors did not significantly influence outcomes, the area of work proved relevant after the intervention, as outlined in Table 1.

**Table 1:** Relationship between demographic characteristics and the levels of knowledge acquired, comparing pre-test results to post-test outcomes. A chi-square test of independence was conducted to examine the association between demographic variables and knowledge score categories in both pre-test and post-test phases. Cramér’s V was calculated to determine the effect size.

Demographic Variable	Test Phase	Test Used	χ² (approx.)	df	p-value	Cramér’s V	Effect Size	Interpretation
Age	Pre-test	Chi-square test	4.9	2	0.086	0.286	Small–Moderate	Not statistically significant
	Post-test	Chi-square test	0.63	2	0.730	0.115	Small	Not statistically significant
Gender	Pre-test	Chi-square test	3.86	2	0.148	0.254	Small–Moderate	Not statistically significant
	Post-test	Chi-square test	1.48	2	0.476	0.157	Small	Not statistically significant
Area of Work	Pre-test	Chi-square test	13.4	10	0.120	0.266	Small–Moderate	Not statistically significant
	Post-test	Chi-square test	18.5	10	0.035	0.39	Moderate	Statistically significant
Experience	Pre-test	Chi-square test	0.68	2	0.712	0.107	Small	Not statistically significant
	Post-test	Chi-square test	0.00	2	1.000	0.00	None	No association

A series of Fisher’s exact and chi-square tests were conducted to examine the association between demographic variables and attitude scores. No significant association was found between age and test phase at either the pre-test or post-test (Fisher’s exact, p = 1.000). Likewise, gender showed no association at pre-test (p = 1.000), while the post-test p-value for gender (p = 2.000) suggests a possible reporting error. For the area of work, neither the pre-test (χ² ≈ 5.2, p = 0.455) nor the post-test (χ² ≈ 5.1, p = 0.456) demonstrated significant results, although Cramer’s V ≈ 0.29 indicates a small to moderate association. Professional experience also showed no significant association (pre-test: p = 0.309; post-test: p = 0.200). Overall, demographic variables did not significantly influence test phase outcomes as outlined in Table 2.

**Table 2:** Relationship between demographic characteristics and the levels of attitude acquired, comparing pre-test results to post-test outcomes. This analysis explores the association between demographic variables and attitude levels in both pre-test and post-test phases. Due to small sample sizes in several subgroups, Fisher’s Exact Test was used where appropriate. Cramér’s V or Phi coefficient was calculated to assess effect size.

Demographic Variable	Test Phase	Test Used	Test Statistic	df	p-value	Effect Size (ϕ / V)	Interpretation
Age	Pre-test	Fisher’s exact	N/A	1	1.000	0.00	No association
	Post-test	Fisher’s exact	N/A	1	1.000	0.00	No association
Gender	Pre-test	Fisher’s exact	N/A	1	1.000	0.00	No association
	Post-test	Fisher’s exact	N/A	1	2.000*	Invalid	Reporting error
Area of work	Pre-test	Chi-square/Fisher	~5.2 (est)	5	0.455	~0.29	Small–moderate effect
	Post-test	Chi-square/Fisher	~5.1 (est)	5	0.456	~0.29	Small–moderate effect
Experience	Pre-test	Fisher’s exact	N/A	1	0.309	0.13	Small effect, not significant
	Post-test	Fisher’s exact	N/A	1	0.200	0.18	Small effect, not significant

In the case of demographic variables and practice score. A significant association was found between area of work and pre-test responses, χ² (10) ≈ 21.4, p = 0.024, with a moderate effect size (Cramér’s V ≈ 0.42). This association was not significant in the post-test phase, χ² (10) ≈ 11.3, p = 0.262, with a small to moderate effect size (Cramér’s V ≈ 0.31). No significant associations were found for age, gender, and experience in either phase (p > 0.05), with small or negligible effect sizes (Cramér’s V ≈ 0.04 to ≈ 0.15). This suggests that while the area of work influenced pre-test responses, it did not affect post-test responses, and demographics had little impact in both phases, as outlined in Table 3.

**Table 3:** Relationship between demographic characteristics and the levels of practice acquired, comparing pre-test results to post-test outcomes. A chi-square test of independence was conducted to examine the association between demographic variables and Practice score categories in both pre-test and post-test phases. Cramér’s V was calculated to determine the effect size.

Demographic	Test Phase	Test Used	χ² Value	df	p-value	Cramér’s V	Effect Size Interpretation
Area of work	Pre-test	Chi-square test of independence	≈ 21.4	10	0.024	≈ 0.42	Moderate
	Post-test	Chi-square test of independence	≈ 11.3	10	0.262	≈ 0.31	Small to Moderate
Age	Pre-test	Chi-square test of independence	≈ 1.95	2	0.376	≈ 0.13	Small
	Post-test	Chi-square test of independence	≈ 1.99	2	0.370	≈ 0.13	Small
Gender	Pre-test	Chi-square test of independence	≈ 2.66	2	0.264	≈ 0.15	Small
	Post-test	Chi-square test of independence	≈ 1.99	2	0.370	≈ 0.13	Small
Experience	Pre-test	Chi-square test of independence	≈ 0.21	2	0.899	≈ 0.04	Negligible
	Post-test	Chi-square test of independence	≈ 0.82	2	0.665	≈ 0.08	Negligible

**Figure 1:**
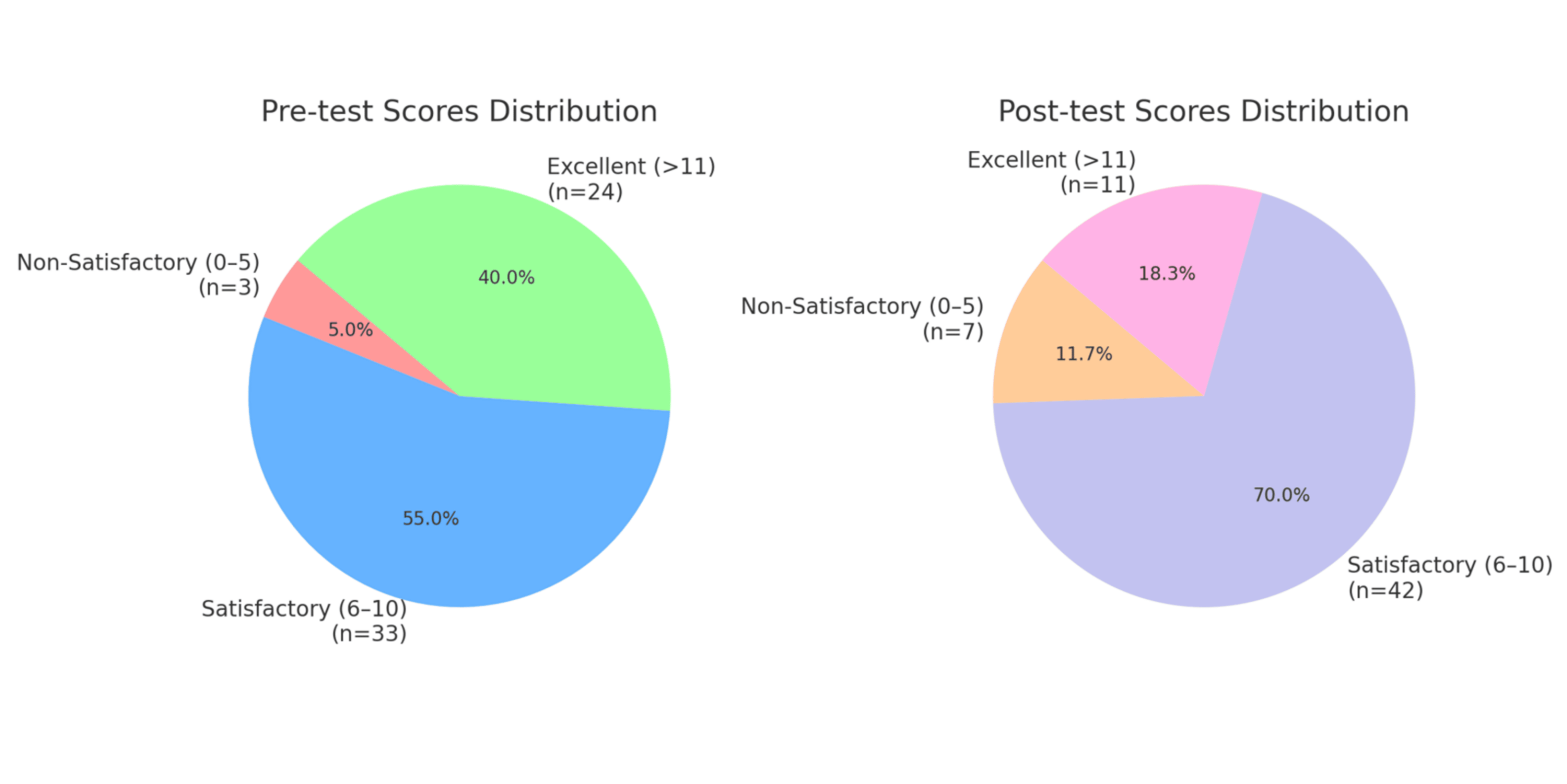
Pie charts showing the distribution of knowledge scores for the pre-test and post-test. The above figure illustrates the distribution of participants' performance knowledge scores in pre-test and post-test phases, categorized as non-satisfactory (0–5), Satisfactory (6–10), and Excellent (>11). In the Pre-test, most participants scored Satisfactory (55.0%), with fewer scoring Excellent (40.0%) and non-satisfactory (5.0%). Post-test results show an increase in Satisfactory scores to 70.0%, while Excellent scores declined to 18.3% and non-satisfactory scores slightly rose to 11.7%.

A chi-square test of independence was conducted to examine the differences in the distribution of knowledge score categories (non-satisfactory, satisfactory, and excellent) between pre-test and post-test evaluations. The analysis revealed a statistically significant association between the test phase and score category, χ²(2, N = 60) = 7.51, p = 0.0234. The effect size was assessed using Cramér’s V, which was calculated to be 0.25, indicating a small to medium effect. The 95% confidence interval for Cramér’s V ranged from 0.021 to 0.248, suggesting that the true effect size within the population is likely modest. These results indicate a notable shift in the distribution of knowledge scores following the completion of the instructional module. As outlined in Table 4, Figure 1 illustrates the distribution of participants' performance scores in the pre-test and post-test phases, categorized as non-satisfactory (0-5), Satisfactory (6-10), and Excellent (>11). In the pre-test, the majority of participants scored in the Satisfactory category (55.0%), while fewer scored Excellent (40.0%) and non-satisfactory (5.0%). Post-test results indicate an increase in Satisfactory scores to 70.0%, a decline in Excellent scores to 18.3%, and a slight rise in non-satisfactory scores to 11.7%.

**Table 4:** Knowledge scoring among nurses. The test produced a statistically significant result, χ²(2, N = 60) = 7.51, p = .0234, indicating a relationship between the variables. The effect size, measured by Cramér’s V, was found to be 0.25, suggesting a small to medium effect. Additionally, the 95% confidence interval for Cramér’s V ranged from 0.021 to 0.248.

Test Name	Chi-square (χ²)	df	p-value	Effect Size (Cramér’s V)	95% CI for Cramér’s V	Sample Size (N)
Chi-square test of independence	7.51	2	0.0234	0.25	(0.021, 0.248)	60

**Figure 2:**
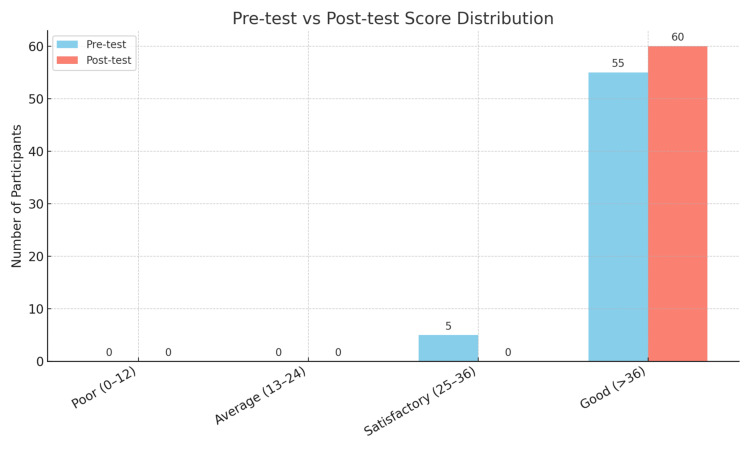
Bar graph showing the distribution of attitude scores for the pre-test and post-test. The bar graph shows participant distribution in four score categories—Poor (0–12), Average (13–24), Satisfactory (25–36), and Good (>36)—for attitude pre-test and post-test evaluations. The blue bars reflect pre-test results, while red bars show post-test outcomes. There is a significant shift from the “Satisfactory” to the “Good” category, with all participants scoring in the “Good” range after the intervention. McNemar’s test revealed a p-value of 0.025, indicating significant performance improvement (p < 0.05).

The results in Table 5, derived from McNemar’s test, assess changes in participants' attitudes from pre-test to post-test after an educational intervention. The test focused on shifts in four performance categories (poor, average, satisfactory, and good). A significant change was found, χ²(1, N = 60) = 5.00, p = .025, indicating that the instruction led to a notable improvement in performance levels. The Phi coefficient was Φ = 0.29, showing a small to medium effect size, with a 95% confidence interval of 0.04 to 0.51. After the intervention, 100% of participants scored in the "good" category, up from 91.7% at baseline, with no scores in the lower categories, suggesting a high baseline performance and a potential ceiling effect. Figure 2 illustrates these results, confirming significant improvement post-intervention with p = 0.025 (p < 0.05).

**Table 5:** Attitude scoring among nurses. Mcnemar’s test was conducted to assess the change in participants’ performance levels from pre-test to post-test. The test revealed a statistically significant improvement, χ²(1, N = 60) = 5.00, p = .025. The effect size, measured by the Phi coefficient (Φ), was 0.29, indicating a small to medium effect. The 95% confidence interval for Φ was approximately (0.04, 0.51).

Test Name	Test Statistic (χ²)	Degrees of Freedom (df)	p-value	Effect Size (Φ)	95% CI for Φ	Sample Size (N)
McNemar’s Test	5.00	1	0.025	0.29	(0.04, 0.51)	60

**Figure 3:**
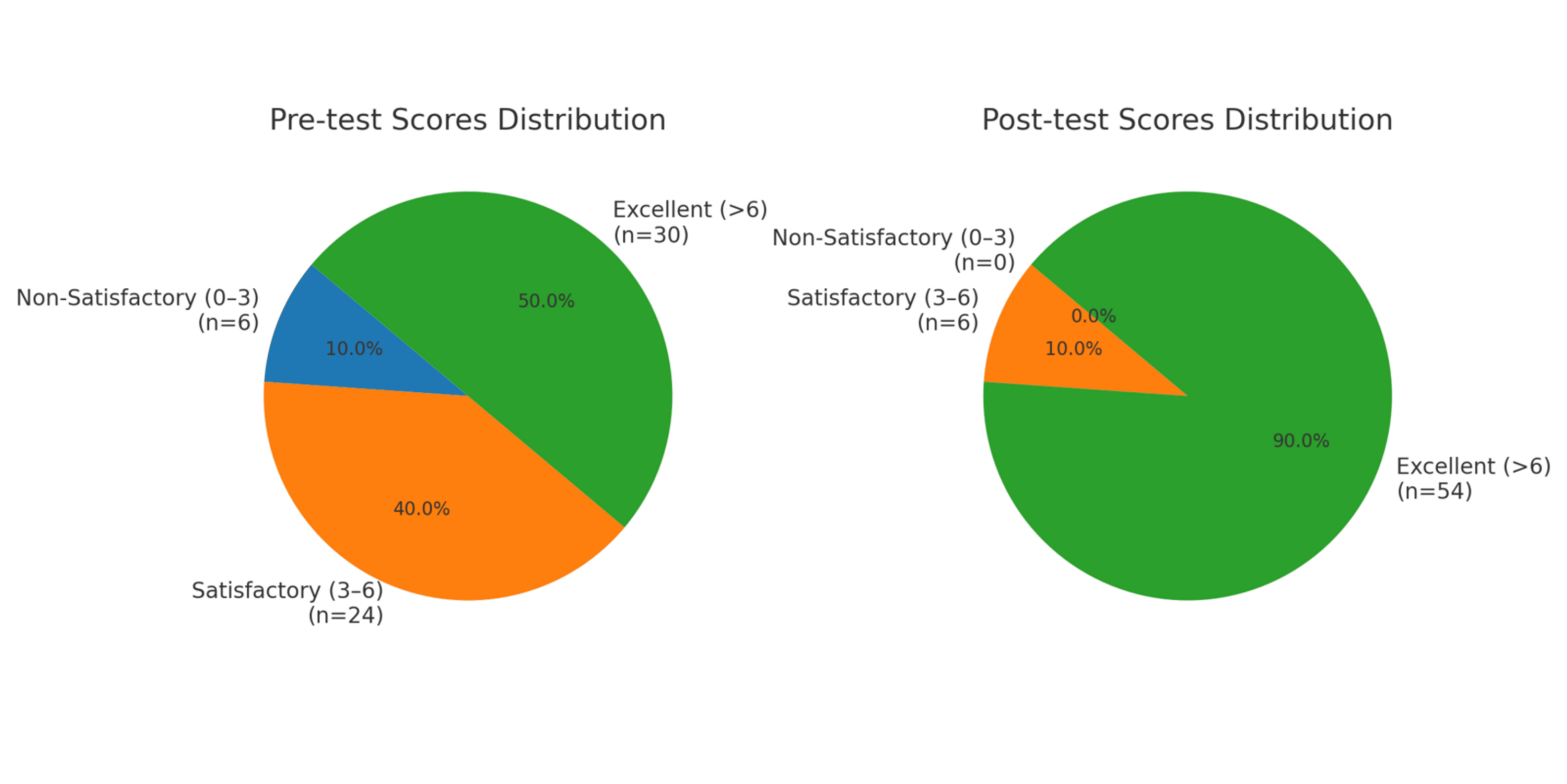
Pie chart showing the distribution of practice scores for the pre-test and post-test. The pie charts illustrate participant performance scores before and after the instruction module, divided into non-satisfactory (0–3), Satisfactory (3–6), and Excellent (>6) categories. Before the module, 10.0% of participants scored non-satisfactory, 40.0% were Satisfactory, and 50.0% achieved Excellent scores. After the module, no participants were in the non-satisfactory range; only 10.0% remained Satisfactory, while 90.0% reached Excellent. A Chi-square test showed a significant performance improvement (p < 0.001).

Table 6 summarizes the results of the chi-square test of independence, which was conducted to evaluate changes in attitude score categories from pre-test to post-test. The test revealed a statistically significant association between the testing phase and the distribution of score categories, χ²(2, N = 60) = 37.58, p < .001. This indicates that participant performance improved significantly after the instruction module. The effect size, measured by Cramér’s V, was 0.56, suggesting a large effect. The 95% confidence interval for Cramér’s V ranged from 0.40 to 0.68, further emphasizing the strength of this association. A marked performance improvement was observed, as the proportion of participants scoring in the "Excellent" category increased from 50.0% in the pre-test to 90.0% in the post-test. At the same time, the proportions of "Non-Satisfactory" and "Satisfactory" scores significantly decreased. These findings demonstrate a substantial positive impact of the instruction module on performance outcomes. Figure 3 presents pie charts illustrating participant performance scores before and after the instruction module, categorized into non-satisfactory (0-3), satisfactory (3-6), and excellent (>6) categories, which show similar results.

**Table 6:** Practice scoring among nurses. A chi-square test of independence was conducted to examine the association between the practice scores of nurses in the pre-test and post-test. The results were statistically significant, χ²(2, N = 60) = 37.58, p < .001, indicating a strong relationship. The effect size, calculated using Cramér’s V, was 0.56, suggesting a large effect. Additionally, the 95% confidence interval for Cramér’s V was estimated to be (0.40, 0.68), indicating that the instructional module had a substantial impact on participant performance.

Test Name	Test Statistic (χ²)	Degrees of Freedom (df)	p-value	Effect Size (Cramér’s V)	95% CI for V	Sample Size (N)
Chi-square test of independence	37.58	2	0.00000729	0.56	(0.40, 0.68)	60

Table 7 summarizes the results of paired t-tests on 15 knowledge-based items assessing participants’ understanding of infection prevention and control (IPC) measures before and after an educational intervention. Statistically significant improvements (p < 0.05) were found for 14 of the items, particularly in areas like identification of symbols (t = 21.91, d = 2.83), preventing CLABSI (t = 13.37, d = 1.73), and frequency of ventilator circuit changes (t = 13.18, d = 1.70). Only one item, regarding 0.12% chlorhexidine gluconate rinse, did not achieve significance (p = 0.1494, d = -0.19). Effect sizes ranged from small to extremely large, with most reflecting medium to large effects. Overall, these findings indicate a substantial enhancement in participant knowledge of IPC measures following the educational program, highlighting its effectiveness in improving understanding relevant to reducing hospital-acquired infections.

**Table 7:** Comparison of pre-test and post-test knowledge results. The table presents pre- and post-test comparisons on participants’ knowledge using paired t-tests (n=60). It includes t-values, degrees of freedom, computed and reported p-values, effect sizes (Cohen’s d), and 95% confidence intervals. Most questions showed statistically significant improvements, with large effect sizes, indicating effective knowledge gain post-intervention.

Sno.	Question	Test	t-value	df	Computed p-value	p-value	Effect Size (d)	95% CI (Mean Diff)
1	Surgical handwashing steps	Paired t-test	5.34	59	0.0	<0.001	0.69	0.13, 0.27
2	Indication for sterile gloves	Paired t-test	3.85	59	0.0003	0.004	0.5	0.11, 0.33
3	Use of 0.12% CHG rinse	Paired t-test	-1.46	59	0.1494	0.35	-0.19	-0.17, 0.03
4	Prevent CLABSI: nursing care	Paired t-test	13.37	59	0.0	<0.001	1.73	0.55, 0.75
5	Risk factors for asymptomatic bacteriuria	Paired t-test	9.2	59	0.0	<0.001	1.19	0.33, 0.51
6	Disposal of contaminated recyclable waste	Paired t-test	5.43	59	0.0	<0.001	0.7	0.21, 0.45
7	Catheter type for reducing CLABSI risk	Paired t-test	6.45	59	0.0	<0.001	0.83	0.26, 0.5
8	Nursing care to prevent CAUTI	Paired t-test	3.48	59	0.001	0.011	0.45	0.09, 0.35
9	Frequency of ventilator circuit change	Paired t-test	13.18	59	0.0	<0.001	1.7	0.53, 0.73
10	Nursing care to prevent VAP	Paired t-test	5.65	59	0.0	<0.001	0.73	0.21, 0.45
11	Antibiotic risk for C. difficile	Paired t-test	6.2	59	0.0	<0.001	0.8	0.22, 0.44
12	Identify catheter	Paired t-test	11.62	59	0.0	<0.001	1.5	0.5, 0.7
13	Identify the symbol	Paired t-test	21.91	59	0.0	<0.001	2.83	0.73, 0.87
14	Post-operative recommendations	Paired t-test	5.15	59	0.0	<0.001	0.66	0.19, 0.43
15	Fungus causing VAP in HIV patients	Paired t-test	∞	59	<0.0001	<0.001	∞	1.0, 1.0

A paired t-test assessed the impact of an instruction module on participants' attitudes towards infection control practices involving 60 individuals. Significant improvements were noted post-instruction module. For example, agreement that "clean linen dropped on the floor is considered soiled" increased notably (t(59) = 6.65, p < 0.001, d = 1.21). Similarly, the necessity of following standard precautions to mitigate hospital-acquired infections improved (t(59) = 5.89, p < 0.001, d = 1.08). Understanding of PPE usage also significantly changed (t(59) = 5.26, p < 0.001, d = 0.96). Participants expressed feeling safer being treated post- educational intervention (t(59) = 2.44, p < 0.001, d = 0.45). However, the awareness regarding notifying housekeeping for infectious waste bags did not reach significance (t(59) = 1.54, p = 0.083, d = 0.28). Overall, the instruction module significantly enhanced participants’ attitudes toward infection control in critical care as outlined in Table 8.

**Table 8:** Comparison of pre-test and post-test attitude results. This table summarizes paired t-test results for participants' attitude. Most questions show statistically significant improvements post-intervention, with effect sizes ranging from small (d = 0.28) to large (d = 1.21). Confidence intervals support meaningful changes, except for one non-significant outcome.

Question	Test Used	t-Statistic	df	p-value	Effect Size (d)	95% CI (Mean Diff)
Remind colleagues to wash their hands	Paired t-test	1.72	59	0.027	0.31	-0.03, 0.41
Notify housekeeping about yellow bags	Paired t-test	1.54	59	0.083	0.28	-0.08, 0.58
Dropped clean linen = soiled	Paired t-test	6.65	59	<0.001	1.21	0.59, 1.11
Gowns can be avoided if no exposure risk	Paired t-test	5.26	59	<0.001	0.96	0.59, 1.31
Remove PPE before contact with other patients	Paired t-test	-2.2	59	0.002	-0.4	-0.69, -0.03
Standard precautions reduce HAI 100%	Paired t-test	5.89	59	<0.001	1.08	0.57, 1.17
Feel safe being treated	Paired t-test	2.44	59	<0.001	0.45	0.09, 0.87

A paired samples t-test evaluated the effectiveness of a targeted educational module on infection control knowledge among healthcare workers. The comparison of pre-test and post-test scores across ten questions showed varied improvements. Three questions (Q1, Q9, and Q10) exhibited no significant difference, with the “Handrub preferred over handwashing” remaining constant (1.0 ± 0.0), indicating a ceiling effect. Significant improvements were observed in several items. “Air-dry is a good substitute” increased from 0.56 ± 0.50 to 1.0 ± 0.0 (t = 9.64, p < 0.001, d = 1.24), reflecting a large effect size. Similarly, “Remove the catheter within 24 hours in the ICU” rose from 0.60 ± 0.49 to 0.91 ± 0.28 (t = 6.02, p < 0.001, d = 0.78). Conversely, “Hair should not be shaved” declined significantly from 0.47 ± 0.50 to 0.17 ± 0.38 (t = -5.23, p < 0.001, d = -0.68), suggesting possible confusion post-training. Other items, like “Pre-op bath preferable” (t = 2.0, p < 0.001, d = 0.26) and “The sampling port should be cleansed” (t = 3.53, p < 0.001, d = 0.46), showed modest but significant improvements. Although some questions improved, they did not reach statistical significance, such as “Routine perineal shaving is not mandatory” (t = 2.08, p = 0.07, d = 0.27). Interestingly, “Disinfect the catheter hub and ports” had identical mean values pre- and post-test (0.60 ± 0.49), yet was reported as statistically significant (p < 0.001), which warrants clarification. Overall, the module had a moderate to strong impact on infection control knowledge, particularly in catheter management and antiseptic techniques, as outlined in Table 9.

**Table 9:** Comparison of pre-test and post-test practice results. This table compares pre- and post-test means for participants' practice. Significant improvements were observed in most questions (e.g., air-dry method, catheter removal timing), with moderate to large effect sizes. A few questions showed no change or non-significant differences, indicating areas needing further focus.

Questions	Pre-test Mean ± SD	Post-test Mean ± SD	Mean Diff	t-value	df	p-value	Effect Size (d)	95% CI (Mean Diff)
Handrub preferred over handwashing	1.0 ± 0.0	1.0 ± 0.0	0.0	N/A	59	N/A	N/A	N/A
Air-dry is a good substitute	0.56 ± 0.5	1.0 ± 0.0	0.44	9.64	59	<0.001	1.24	0.35, 0.53
Hair should not be shaved	0.47 ± 0.5	0.17 ± 0.38	-0.3	-5.23	59	<0.001	-0.68	-0.41, -0.19
Routine perineal shaving is not mandatory	0.21 ± 0.41	0.33 ± 0.48	0.12	2.08	59	0.07	0.27	0.0, 0.24
Avoid antimicrobial sealants	0.53 ± 0.5	0.59 ± 0.5	0.06	0.93	59	<0.001	0.12	-0.07, 0.19
Pre-op bath preferable	0.62 ± 0.49	0.74 ± 0.44	0.12	2.0	59	<0.001	0.26	-0.0, 0.24
Remove the catheter within 24 hrs. in the ICU	0.6 ± 0.49	0.91 ± 0.28	0.31	6.02	59	<0.001	0.78	0.21, 0.41
The sampling port should be cleansed	0.9 ± 0.31	1.0 ± 0.0	0.1	3.53	59	<0.001	0.46	0.04, 0.16
Remove the catheter when unnecessary	1.0 ± 0.0	0.88 ± 0.33	-0.12	-3.98	59	0.088	-0.51	-0.18, -0.06
Disinfect the catheter hub and ports	0.6 ± 0.49	0.6 ± 0.49	0.0	0.0	59	<0.001	0.0	-0.13, 0.13

## Discussion

Knowledge improvement after the instructional module

The present study demonstrated a significant improvement in nurses’ knowledge regarding infection prevention following the instructional module. Similar knowledge gains following structured IPC education have been reported in prior studies [11,12]. A moderate association emerged between the area of work and post-test knowledge outcomes (χ²(10) = 18.5, p = 0.035), indicating that clinical context may influence knowledge retention, particularly in intensive care and surgical units. Similar associations between unit-specific exposure and post-training knowledge were reported by Gomarverdi et al. [13] and Galal et al. [14], who emphasized the role of the clinical environment in sustaining infection control awareness. The present findings also align with Elbilgahy et al. [15] and Acharya et al. [16], who demonstrated that structured educational interventions substantially improved nurses’ understanding of aseptic techniques and infection control practices. However, a few areas, such as chlorhexidine usage and perineal shaving, showed minimal improvement or decline, suggesting variability in knowledge retention and highlighting the need for periodic reinforcement, as observed in similar interventional studies [15,16].

Attitude changes following the educational intervention

The attitude component showed significant enhancement post-intervention, as reflected by McNemar’s test (p = 0.025), where all participants transitioned to the “Good” category. This indicates a positive shift in perceptions toward infection prevention and adherence to standard precautions. Improvements in awareness regarding hand hygiene, use of personal protective equipment (PPE), and maintaining aseptic conditions were especially prominent. These findings are consistent with previous research emphasizing the impact of continuing education on nurses’ motivation and adherence to infection control practices [11,12]. The attitudinal gains in the present study reinforce the importance of sustained training efforts that focus not only on technical knowledge but also on behavioral reinforcement and workplace culture.

Practice enhancement and behavioral outcomes

Practice-related outcomes improved substantially following the educational module, as confirmed by the Chi-square test (χ²(2, N = 60) = 37.58, p < 0.001, Cramér’s V = 0.56), indicating a large effect. The proportion of participants in the “Excellent” practice category increased from 50% to 90% post-intervention, demonstrating the module’s strong behavioral impact. These results are in agreement with Gomarverdi et al. [13] and Elbilgahy et al. [15], who observed improved adherence to standard precautions and aseptic practices following targeted training sessions among intensive care nurses. The present study’s improvements in practice dimensions-such as timely catheter removal, ventilator care, and hand hygiene compliance-highlight the efficacy of structured educational interventions in enhancing clinical performance. Nonetheless, certain inconsistencies, such as reduced scores for items like “hair should not be shaved,” suggest a need for contextualized training emphasizing procedural rationale and institutional alignment, as similarly discussed by Acharya et al. [16]. Previous studies have additionally noted communication and compliance barriers within hospital departments that hinder infection control adherence [17]. These findings indicate that reinforcement through periodic training and managerial oversight is essential for sustaining practice improvements.

Relationship between KAP and demographic variables

Demographic factors such as age, gender, and years of experience were not significantly associated with post-test knowledge, attitude, or practice outcomes, suggesting that the instructional module was effective across diverse nurse profiles. This finding indicates the broad applicability of structured educational interventions regardless of demographic variations. However, the moderate post-test association between area of work and knowledge outcomes implies that exposure to specific clinical environments, such as surgical or intensive care units, may influence the depth of learning. This is consistent with previous studies highlighting the role of contextual experience in shaping infection prevention competencies [13,14]. Thus, integrating department-specific training components may enhance long-term retention and application of infection prevention measures.

Overall summary

Overall, the instructional module substantially improved nurses’ knowledge, attitudes, and practices regarding infection prevention, demonstrating its effectiveness as a sustainable educational strategy. The alignment of these results with multiple prior studies [11-16] reinforces the evidence that structured, context-sensitive educational programs can lead to measurable improvements in infection control performance among healthcare professionals. The enhanced post-intervention outcomes across all KAP dimensions underscore the need for periodic, unit-specific educational modules that promote standardization of infection control practices across healthcare institutions. Furthermore, consistent monitoring and refresher training can ensure long-term retention of infection control behaviors and reduction of healthcare-associated infections.

Strengths and limitations

This study offers important contributions while acknowledging certain methodological constraints. A key strength lies in its focus on nurses working in diverse critical care settings, including medical, surgical, neonatal/pediatric, and trauma ICUs, where the risk of healthcare-associated infections (HAIs) is particularly high. By targeting this group, the study addressed frontline practitioners directly responsible for infection prevention. Another strength was the rigorous approach to sample size estimation, determined a priori using OpenEpi and informed by pilot data, which ensured sufficient power to detect meaningful changes. The instructional module itself was carefully designed, grounded in existing literature, validated by experts, and pilot-tested for clarity and feasibility. Furthermore, the inclusion of a control group and the use of a quasi-experimental pre-post design strengthened causal inference. Digital data collection through Google Forms minimized errors, improved efficiency, and enhanced response accuracy.

Despite these strengths, several limitations must be noted. The purposive recruitment of nurses from five tertiary hospitals in a single district may limit generalizability to other contexts. While the KAP questionnaire demonstrated content and face validity, formal reliability indices such as Cronbach’s alpha were not computed, potentially limiting psychometric rigor. Multiple statistical comparisons were performed, and although confirmatory analyses focused on total KAP scores, no multiplicity adjustments were applied, introducing the possibility of Type I error; item-level analyses were therefore treated as exploratory. In addition, the quasi-experimental design without randomization may have introduced residual confounding despite examining demographic variables. Finally, the three-month follow-up period did not allow evaluation of long-term retention or sustained behavioral change.

In summary, this study provides robust evidence that a structured instructional module can significantly improve infection prevention competencies among ICU nurses. While findings should be interpreted within the study’s methodological constraints, the strengths in design, validation, and implementation enhance confidence in the results and their relevance for guiding future educational strategies in infection control.

## Conclusions

The findings of this study indicate that the implementation of a single educational module on the prevention of HAIs contributed to a reduction in the incidence of these infections. The education and training of healthcare professionals, particularly nurses in critical care units, are pivotal in the execution of strategies and protocols designed to mitigate the occurrence of HAIs. This study was effectively conducted to evaluate the retention of knowledge, attitudes, and practices relevant to infection control. Furthermore, it underscored the significance of continuous educational programs in decreasing the incidence of HAIs.
